# Diagnostic radiographers' perceptions of professional identity in Johannesburg, Gauteng, South Africa

**DOI:** 10.1002/jmrs.737

**Published:** 2023-11-10

**Authors:** Keleabetswe Mtombeni, Lynne Hazell, Louisa Mokoena

**Affiliations:** ^1^ Department of Medical Imaging and Radiation Sciences, Faculty of Health Sciences University of Johannesburg Rooderpoort South Africa

**Keywords:** Healthcare, identities, Professional, radiographers

## Abstract

**Introduction:**

The study explored and described the professional identity of diagnostic radiographers in Johannesburg, Gauteng, South Africa.

**Methods:**

The methodology employed for the study was qualitative, exploratory and descriptive design. Face‐to‐face interviews were conducted with thirteen diagnostic radiographers from private, public practices and individually owned practices. Semi‐structured interviews were conducted, and the responses underwent thematic analysis and used Braun and Clarke's six steps for analysing qualitative data.

**Results:**

The thematic analysis revealed three themes and six categories. The three themes identified were: perceptions of identity, environment influences and organisational institutions.

**Conclusion:**

This study provided an insight into the current perspectives of the professional identity of diagnostic radiographers in Johannesburg, South Africa, which reflected a positive professional identity. The three themes identified could inform guidelines for education in diagnostic radiographers' professional identity in the future.

## Graphical abstract

The article is about the positive professional identity of diagnostic radiographers from Johannesburg, South Africa. There are various negative factors that affect their professional identity; however, they continue to love their profession.

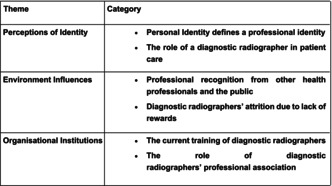

## Introduction

Professional identity is a concept that explains how we perceive ourselves within our occupational context and how we communicate this to others.^1^ Professional identity develops in collusion with the individual's personal identity.^2^ Personal identity is intangible and results from individuals' perception of themselves in their profession of choice.^2^ Furthermore, individual personal identity is constructed of social influences, personal identity and ego identity.^2^ Therefore, our personal and professional identities are not separated; rather some identities are in the foreground while others are in the background, all these depend on the context.^3^

People have multiple identities, and healthcare workers are likely to identify as being part of a multidisciplinary team; furthermore, they form part of the organisation as an employee or as a member of a professional board.^4^ Additionally, defining feature of professional identity in the healthcare sector is the fundamental commitment to caring.^5^ An example of this is noted with the need to change people's lives, to improve them.^5^ The fundamental commitment to helping is supported by a professional stance which includes the aggregation of knowledge and skills.^5^

Stalsberg and Thingnes in their study defined the professional identity of a Norwegian radiographer as being dual in nature.^6^ This duality arises from the notion that radiographers work with both scientific mechanical technology and patient‐centred care to which they must apply their humanistic nature.^6^ In the South African context, the Scope of Practice identifies that radiographers should accurately use technology for the production of images and that they work in collaboration with other health professions and consider the well‐being of the patient.^7^ There is a similarity in the role of the radiographer in both countries; however, how does this impact on their perception of their professional identity?

South African radiographers may be employed in the public sector, the private sector or have their own practice. From this, it can be noted that the professional identity of diagnostic radiographers may vary due to the context in which they practice. In addition, diagnostic radiographers have various other professional roles such as application specialists, managers of departments and clinical tutors. This research aimed to explore and describe the perceptions of professional identity of diagnostic radiographers based in Johannesburg, South Africa.

## Population

The population were diagnostic radiographers who were independently registered with the Health Profession Council of South Africa (HPCSA). There was a population of 3082 diagnostic radiographers in Gauteng in 2019.^8^ There are different categories of registration with the HPCSA. Initially, students are registered with the HPCSA as a student radiographer.^9^ After graduation, 1 year of community service is compulsory. Once completed, graduates are registered as independent radiographers.^9^ In South Africa, there training of radiography is at eight universities across the country. Prior to 2014, a National Diploma in diagnostic radiography was taught by most universities. Since 2014, the universities have been teaching a 4‐year degree in diagnostic radiography.

The researcher also used purposive and snowball sampling enabling radiographers to introduce other participants to the researcher. Thirteen diagnostic radiographers were interviewed (Table 1), six radiographers were working in the public sector, four radiographers were working in the private sector while three radiographers owned their own practices.

**Table 1:** Overall‐overview of the population sample used in this study.

Description	Category	Number
Gender	Male Female	7 6
Number of years experience	1–5 years 5–10 years 10–15 years 15–20 years 20–30 years	3 5 3 0 1
Employment sector	Public sector Private sector Own a practice	6 4 3
Qualification	Ndip diagnostic radiography B‐Tech diagnostic Mammography Other qualification	13 5 4 2
Age	20–29 years 30–39 years 40–49 years 50–59 years 60–70 years	3 6 2 1 1
Total number of participants		13

There is a vast difference noted between diagnostic radiographers working in private hospitals versus those in public hospitals. Diagnostic radiographers in private hospitals are well compensated and experience no budgetary constraints in carrying out their daily duties.^10^ In the public sector, diagnostic radiographers are compensated in accordance with the Occupation Specific Dispensation (OSD).^13^ Furthermore, diagnostic radiographers are paid amounts according to the number of years they have been qualified.^10^

The National Department of Health (NDOH) permits diagnostic radiographers to open their own private practices.^11^ Thus, diagnostic radiographers are one of the selected few professions that are allowed to open their own practices.^11^ This study also included diagnostic radiographers who own their own private practices. Certain diagnostic radiographers who owned their own private practices, worked as sessional diagnostic radiographers in public institutions as well.

## Methodology

Ethical approval was obtained from the University of Johannesburg research ethics committee (REC 241112‐035). The methodology employed for this study was a qualitative, exploratory, descriptive design. Semi‐structured face‐to‐face interviews were conducted with independent diagnostic radiographers to provide information‐rich accounts of their perceptions of radiographers' professional identity. Diagnostic radiographers in Gauteng have multiple realities in their professional environment and interact differently with various role players in the health‐care field.

The researcher gained permission from the public and private hospitals to conduct research once ethical approval was granted. The researcher then personally approached the head of department of the radiology departments to communicate the purpose of the study. Information letters and consent forms were provided. Information letters and consent forms were provided to the heads of department. Diagnostic radiographers who wished to take part in the research signed consent before interviews could be conducted. Due to COVID 19 protocols interviews were conducted at the research participant's homes, at restaurants or outside their workplace during their lunch breaks.

Individual audio recorded interviews were conducted, and the interview time ranged from 20 to 50 min. All thirteen interviews were done within a period of 3 months. All interviews started with a broad question about the participants understanding of professional identity, and the follow‐up questions had the participants describing their own personal professional identity. This was followed by additional probing questions from the statements that were provided by the participants or from the interview schedule. The interview process continued until no new information was presented. This state of data saturation was reached after the 13th interview. Data saturation denotes that informational redundancy has been reached since additional data contributes little to the research study.^12^, ^13^

### Data analysis

Thematic analysis was used to analyse the data. Through its theoretical freedom, thematic analysis is highly flexible and can be modified for most studies.^14^ To achieve data analysis Braun & Clarke's six‐phase framework was used (Table 2).^14^, ^15^ Furthermore, inductive analysis was then applied, which allowed the researcher to work back and forth between codes and themes. The researcher developed themes that emerged from the interviews. These themes were discussed with her 2 supervisors whose qualification ranged from a doctorate in radiography to masters in radiography. This was done in order to achieve rigour and validity.

**Table 2:** Detailed summary of Braun & Clarke's 2006 six‐phase framework that was used for data analysis.^15^, ^16^

Step 1 Become familiar with the data. The audio recordings were repeatedly listened to by the researcher. The professionally transcribed data was read repeatedly Step 2 Generate initial codes. Words or paragraphs that stood out from the verbatim statements were highlighted and assigned shorthand labels (codes) Step 3 Search for themes. The coding was focused on a broader level of analysis, which derived themes Step 4 Review themes. Once themes were formed the researcher read through the data, to see if the themes were a correct representation of the data collected Step 5 Define themes. Themes and sub‐themes that were generated during stage three and four were defined. The meaning of each theme was captured and discussed Step 6 Write‐up. Extracts from the transcription were used to demonstrate the transcriber's findings and show a representation of the data collected

Furthermore, methodological triangulation was used. This included semi‐structured interviews, field notes collected during the interviews, and reflexivity where the researcher acknowledge her role as part of the research. All interviews started with diagnostic radiographers explaining what their definition or perception of professional identity is? Field notes allowed the researcher to consider the non‐verbal responses of the radiographers during the interviews and reflexivity enabled the reflection on the role as the researcher and as a diagnostic radiographer.

Lincoln and Guba's 1985 measures for trustworthiness were used.^17^ For this study, credibility was achieved through member checking, peer debriefing, reflexivity, data triangulation and prolonged engagement. A complete description of the research setting, the population and the methodology was described in detail to achieve transferability. To achieve confirmability, all data procedures were kept transparent with the decisions and choices justified. Dependability was achieved through methodological triangulation and member checking.

Ethical approval was obtained from the University of Johannesburg research ethics committee. For this research, participants signed an informed consent form to participate in the interviews and an informed consent for interviews to be audio recorded.

## Findings and Discussion

Thirteen diagnostic radiographers participated in the semi‐structured interviews. From the thematic analysis, three themes emerged and included perceptions of identity, environment influences and organisational institutions, these are listed in Table 3 below and discussed in the paragraphs that follow.

**Table 3:** A detailed table about themes and categories developed in the study.

Theme	Category
Perceptions of identity	Personal Identity defines a professional identity The role of a diagnostic radiographer in patient care
Environment influences	Professional recognition from other health professionals and the public Diagnostic radiographers' attrition due to lack of rewards
Organisational institutions	The current training of diagnostic radiographers The role of diagnostic radiographers' professional association

### Theme 1 Perceptions of Identity

Diagnostic radiographers indicated that their personal identities impacted on how they perceived their professional identity. A definition of the professional identity of a diagnostic radiographer was not easy for the diagnostic radiographers to clarify. When the research diagnostic radiographers were asked what they understood about the term professional identity, many of them divided the term into personal and professional identity.

Okay, firstly, professional identity it's the perception that other professions that we work with – ja, and in association with, we work together and how they look at us, and at the end of the day the values that I have also about myself, the view that I have for myself. You know, because I believe identity it is a two‐way thing, either from the point of the person that we interact with and also from my point of view. Interview 1: Private sector

When I look at the “word” itself like profession and the identity, I can say the way I identify or relate myself like with other people, that can be my colleagues, that can be my patient or other stakeholder in the hospital and the way I relate to them. And looking also at myself, like let‟s say, “self” point of view as well. Myself and also the way I see others and the way they also see me, but in a professional way. Interview 4: Public sector

In addition, the diagnostic radiographers highlighted that who they are as individuals is very important. They added that their personal identities give their lives purpose, meaning, guides their behaviour and influence their professional identities. The diagnostic radiographers argued that it is hard to differentiate between their professional identity and personal identity. They highlighted the brevity required to adapt their personal identities depending on the department that they are allocated to at work.

So for me I feel like I need to have a certain personality every time when I walk into the work environment, right, and then I need to change my personality according to the environment or the department that I will be placed in on the day. Interview 9: Public sector

It is very hard. It is very hard to differentiate between the person I am and the person I want to be in a department. I personally feel that I am “soft‐spoken‟, but in my department it become frustrating when things don′t work accordingly. Interview 5: Public sector

The brevity of a diagnostic radiographer's personal identity is subjective and is determined by who they are and the work environment that they are allocated to. Diagnostic radiographers have multiple identities, which is dependent on the environment that they are working in. The diagnostic radiographers who were interviewed demonstrated the multidimensional nature of professional identity. However, their identities are compartmentalised to a specific context and environment. Similarly, Warren and Braithwaite in their research study about healthcare workers' professional identity states that identities are varied, and multiple identities can be held by one professional.^5^ A pharmacist can have up to nine identities, which can range from a scientist to a businessperson.^5^

Furthermore, the role a diagnostic radiographer has within the healthcare system was very important to those who were interviewed. The areas that they focused on include radiation control, patient‐centred care, communication with patients, and the justification of examinations.^5^, ^18^, ^19^

And working with patients is important, we need to explain to our patients. Patients are scared, they don′t know what to do when they come to a hospital. So we need to be there for them and understand and talk to them. Language is also a problem. In South Africa we all speak multiple languages. So as a radiographer we need to know that we need to “communicate” properly with them. Interview 5: Public sector

Diagnostic radiographers interact with the patient firstly by explaining the procedure and then acquiring the clinical history related to the examination. A number of examinations carried out by the radiographer require the patient not to move. Thus, effective communication is necessary to calm the patient and make them feel comfortable to achieve quality imaging.^20^

I have to take extra caution to make sure that I do it best as possible with the reduction of radiation. Interview 1: Private sector

Diagnostic radiographers are one of the few professionals in the healthcare sector licensed to work with ionising radiation.^21^ Radiographers' responsibilities include history taking, verifying patients' identity, the justification for the procedure to be performed, screening for safety, providing instructions, ensuring that patients understand all instructions and answering questions promptly and accurately.^22^, ^23^ In South Africa (RSA), individuals who are appropriately trained as radiographers or radiologists, and registered with the HPCSA, are licensed to operate X‐ray equipment.^18^

### Theme 2 Environmental Influences

Environmental factors such as professional recognition and the attrition of diagnostic radiographers have influenced the way the current diagnostic radiographers in the study perceive their professional identity.

Professional recognition within the healthcare environment was a dominant category for the diagnostic radiographers who were interviewed. Diagnostic radiographers argued that they experience a lack of professional recognition within the hospital environment. The current COVID‐19 pandemic has heightened these feelings that radiographers are not recognised as frontline workers.

Throughout the pandemic, the diagnostic radiographers felt that only nurses and doctors were recognised as frontline workers and highlighted that diagnostic radiographers were not considered essential workers.^22^ They argued that doctors and nurses received Personal Protective Equipment (PPE); however, diagnostic radiographers did not receive any PPE even though they were examining the same patients. The sentiment is shared by the diagnostic radiographers who were interviewed and is similar to those views expressed in a study regarding diagnostic radiographers’ experience of COVID‐19 in Gauteng, SA.^22^

A very big example is the pandemic during the Covid‐19. First of all we were not regarded as essential workers, although we were at work 24/7. I mean we had allocations put in place that covered the Covid‐19 of how we were supposed to work. Right, so it got so bad that in the Hospital that I work at the nurses would get PPE, but we as radiographers had to buy our own PPE. And what was so awful about this is that the same patients that the nurses are transporting to our X‐ray department, we are dealing with the very same patients that they are wearing PPE for, whereas we don′t have PPE. Interview 9: Public sector

Furthermore, there is a lack of inter‐professional recognition that the diagnostic radiographers experience in their working environments. Diagnostic radiographers feel overlooked and underappreciated.

I feel that they don′t look at us as part of their team. They don′t recognise that X‐rays are important or a radiographer is important. So, when they plan their days, and they say we “need” so many patients for theatre. They don′t say, let's phone X‐rays and see if they are fully staffed. Can they give us a radiographer after hours or during working hours? It's not. It's that they plan their lives and then phone and then phone, we need a radiographer now. Interview 6: Owns practice

These sentiments are similar to that of the participants in a study by Naylor and Foulkes, and there is a lack of clarity about the role of the radiographer and when a radiographer will be needed in theatre and the type of imaging that is being requested.^23^ Diagnostic radiographers are called to operating theatre in the middle of the operations. Failure to communicate with radiographers resulted in them feeling like outsiders.^23^ This made them feel that their profession is not respected.^23^

Diagnostic radiographers who were interviewed highlighted that professional stagnation is another challenged that they are faced with. These sentiments are similar to those expressed in a research study conducted in South Africa conducted by Britton, Pieterse and Lawrence in 2017, where professional fulfilment was not achieved due to professional stagnation and the lack of professional recognition.^10^

They want to grow but they find themselves stuck because of the environment, whether they can't do other specialities like Nuclear Medicine and what. And after a while maybe they may want to go into something else and don't know how or what. Interview 6: Owns practice

The diagnostic radiographers indicated that there is no clear career path, monetary compensation is poor and even when studying further there is a lack of recognition of postgraduate qualifications. Professional recognition has a role to play in the creation of a negative impression of the profession. The radiographers in the study indicated a high workload and a lack of career opportunities and professional development motivated them to consider changing their professions.^24^ The diagnostic radiographers were despondent due to the lack of a career path. Additionally, they do not see a need to increase their knowledge academically as they feel it will not make any difference to their income. There was a sense of melancholy amongst most of the diagnostic radiographers participating in the study when discussing post‐graduate studies.

You can do your master's, you can do your doctorate but then in the field, like you will still be on the floor, you're still limited. You can have your doctorate but then you still do X‐rays the way you did them ten years ago it's not like when you have a doctorate or you have your whatever qualification, you're going to move up. Interview 2: Public sector

Even though there are challenges in the field of diagnostic radiography that might be demoralising to diagnostic radiography professionals. There were several diagnostic radiographers who were interviewed who spoke passionately about the profession and the love that they have for it.

I mean radiography is a deep love. It's a love that I don't think anyone can just fall into. I mean I have started this journey in radiography with people that flunked out in the second year and they're like, no, this is not for me. Interview 11. Owns practice

There are those that left the profession to pursue other ventures, and then came back to the profession and started from the bottom. This demonstrates that diagnostic radiographers may think other careers are more appealing but return to their radiography profession.

So I have been in the field for over 20 years. I have started at the bottom as a normal radiographer. I am in management, but when I was in management. previously I left and I came back. So I started again at the bottom, and I've “re‐climbed” if you have to say that back to management position. Interview 3: Public sector

There were those who have been in the field for more than 40 years demonstrating a love for the profession. The diagnostic radiographer quoted had worked overseas and experienced many changes in the profession.

Okay, I qualified in 1977… I was one of those radiographers that could not sit endlessly in one institution. It was during the apartheid days, which was very difficult for us as non‐white radiographers to move into the field of radiography to other institutions. Interview 8: Public sector

It is clear to see from the above quotations that diagnostic radiographers are very passionate about their jobs, have a positive professional identity, and love working with patients.

### Theme 3 Organisational Institutions

The final theme discusses how organisational institutions form part of the external pressures that affect the formation of a professional identity. Organisational institutions such as professional bodies and academic institutions have a major impact on the individual's professional identity. The diagnostic radiographers who were interviewed discussed how their professional identity is naturally linked to the academic institutions that they graduated from. These institutions represent their individual purpose, values and principles. It is during these academic years that student radiographers are socialised and integrated into the profession. As a result, there is an ongoing process of professional identity construction and deconstruction experienced.^25^

They already know their line, that I am the one that makes the rules. So if you go around as an academic institution where you make sure that the students they know, we are the ones that protect the public against radiation. Interview 1: Private sector

On the other hand, the diagnostic radiographers who owned their own practices felt that the academic institutions did not equip them well enough to open their own practices. There were certain aspects of running their own practices that they had to learn through experience only.

The issue about owning my own practice is another issue in terms of recognition. It's a different thing because there is competition, you know, you're competing with established people. You're competing with people who can easily get finance. So it's a different thing, and I understand because it's business. You know one of the challenges is you're coming as someone who is skilled to do X‐rays, you know, who understands what you are doing in terms of that. But there are many things that come along with it in terms of – which is why I then wished, maybe I should have advanced my studies. But does my studies have the business run of things? You know, at least you learn the designs and other things, but specifically in terms of now you have to know how to, you know, register your people with the department of labour and do your tax and all those things, it gets more complicated. How do you market yourself? Where do you place yourself? How do you even get finance for equipment? Because equipment is very expensive. Interview 6: Owns practice

This quote demonstrates that the undergraduate national diploma course does not equip diagnostic radiographers with the knowledge to open their own practices. This could be addressed in the learning outcomes in a degree programme. There are several business syllabus topics which are particularly pertinent to diagnostic radiographers. These include innovation and entrepreneurship, marketing, economics, accounting and finance, strategy, organisational behaviour and human resource management.

The South African Society of Radiographers (SORSA) is a professional organisation which diagnostic radiographers belong too. SORSA encourages professional development through seminars and CPD programmes. Diagnostic radiographers who were interviewed did not speak passionately about SORSA. In addition, most of the radiographers in the study were not affiliated with SORSA. Diagnostic radiographers believed that SORSA was ineffective, hence why there is a lack of professional recognition and career empowerment.

It is very important for radiographers to have a healthy professional identity. I mean for instance we have a radiography society, SORSA, I think. I think which is to my knowledge is supposed to be empowering us and standing up for us and helping us to get more recognised. Interview 9: Public sector

It is evident that diagnostic radiographers do not get involved with their professional associations, as only 30% of the diagnostic radiographers who participated in this study were registered with SORSA. Being part of a professional organisation encourages the advancement of the profession. Diagnostic radiographers complain about the lack of professional recognition. However, for the profession to be recognised and have a public profile, diagnostic radiographers need to invest in their own professional associations.

## Discussion

Diagnostic radiographer's who were interviewed responded positively when discussing their professional identity. Furthermore, they valued the role that they played in saving patients' lives and interacting with them. However, the participants mentioned the lack of professional recognition by the public, as well as in their interprofessional environment. Diagnostic radiographers exhibited frustrations in the profession due to the lack of recognition of their post‐graduate qualifications and the lack of a career path. This negatively impacts the development of professional identity. As a result, diagnostic radiographers who were interviewed felt discouraged to pursue studying any further in diagnostic radiography.

Despite all these challenges, diagnostic radiographers spoke about their deep love for radiography. No matter how many years diagnostic radiographers were qualified, or the environment that they worked in, they all spoke about their love for radiography positively. In light of the information extracted from the data collected, guidelines were developed that will assist in enhancing environmental factors that affect diagnostic radiographers' professional identity. These include interprofessional learning and investing in their own professional associations.

### Implications for practice

The findings could inform the Professional Board for Radiography and Clinical Technology (PBRCT) about the current professional identity of diagnostic radiographers. The needs of radiographers to develop a professional identity could be addressed by the PBRCT in terms of education and training in the radiography curriculum and continuous professional development activities.

## Limitations

The study was geographically based in Johannesburg, Gauteng, and so excluded diagnostic radiographers across SA and therefore is not representative of the radiography profession in the country. The Corona pandemic had an impact on the current study, as it was difficult to get a large number of participants due certain restrictions of COVID 19.

## Conclusion

This study discovered that diagnostic radiographers do have a positive professional identity; however, there are multiple factors that influence their identity both personal and professional. From the themes factors impacting professional identity include professional recognition and the role of a diagnostic radiographer when interacting with a patient. The lack of professional recognition highlighted in the study could be improved by increased public awareness of the profession and interprofessional collaboration.

## Data Availability

The data that support the findings of this study are available from the corresponding author upon reasonable request.
